# Prenatal maternal infection promotes maternal microchimeric cells to alter infection risk in male offspring

**DOI:** 10.3389/fimmu.2026.1869048

**Published:** 2026-09-03

**Authors:** Brittany Jimena, Daniel Kazimierczyk, Simon Kazimierczyk, Haley Moya, Eva Shin, Li Li, Parimal Korgaonkar, Caryn Porter, Brian Seed, Bobby J. Cherayil, Nitya Jain

**Affiliations:** 1 Mucosal Immunology and Biology Research Center, Massachusetts General Hospital for Children, Charlestown, MA, United States; 2 Lurie Center for Autism, Mass General for Children, Boston MA, United States; 3 Center for Computational and Integrative Biology, Massachusetts General Hospital, Boston, MA, United States; 4 Department of Pediatrics, Harvard Medical School, Boston, MA, United States; 5 Mucosal Immunology and Biology Research Center, Mass General for Children, Charlestown, MA, United States

**Keywords:** maternal microchimeric cells, pregnancy-associated infection, *Salmonella typhimurium*, type 17 effector T cells, vertically transmitted immunity

## Abstract

Vertically transferred maternal cells or maternal microchimeric cells (MMCs) engraft the fetus and persist in offspring for long periods of time. How altered maternal immune states arising from infection affect MMCs and their function in offspring is poorly understood. Here, we show that pregnancy-associated transient maternal infection alters MMCs to differentially regulate immunity in offspring. In male offspring of dams previously infected with *Yersinia pseudotuberculosis*, MMCs confer a pro-inflammatory type 17 T effector phenotype that leads to enhanced protective immunity to an unrelated *Salmonella* infection. Thus, acquired maternal cells imprinted by microbial exposure during pregnancy exert an antigen-agnostic and sex-differential effect on offspring immunity, and may potentially be targeted to deliver immune benefits to infants in the vulnerable early life period.

## Introduction

Exposure to infectious agents during pregnancy can impact offspring immune responses to infections and vaccinations (1). Chronic infection during pregnancy can affect infant health independently of pathogen transmission. For instance, HIV-exposed but uninfected infants have reduced levels of maternal antibodies and show increased susceptibility to infection by unrelated pathogens compared with infants not exposed to HIV (2). Human malarial and helminth infection during pregnancy correlates with impaired immunoglobulin G (IgG) responses to *Haemophilus influenzae* and diphtheria antigens (3). Vertically transferred maternal immunity including antibodies and cytokines have a large influence on immune responses in early life. Maternal cells that engraft the fetus during gestation, called maternal microchimeric cells (MMCs), also have the potential to influence offspring immunity (4).

Acquired MMCs are retained in offspring for long periods of time and are proposed to have both harmful and beneficial roles (5, 6). Alloimmune inflammatory responses targeting genetically disparate MMCs present in heart tissues contribute to the pathogenesis of cardiac injury in neonatal lupus (7). Acquired maternal T cells initiate islet inflammation and predispose to diabetes in an animal model of autoimmunity (8). MMCs contribute to tissue repair (9, 10) and substitute for missing fetal immune components in immune-deficient animals (11, 12). MMCs also regulate offspring immunity through antigen-specific and non-specific mechanisms. Immunodeficient infants with Epstein–Barr virus (EBV) and cytomegalovirus (CMV) infection harbor maternal CD8^+^ T cells that are reactive to EBV and CMV antigens, respectively (13, 14). In an animal model of preconceptual infection with OVA-expressing *Listeria*, OVA-specific maternal T cells are transferred to the fetus during pregnancy (15). The presence of these chimeric T cells in offspring correlates with less severe disease following infectious challenge in early life. In addition to contributing to pathogen-specific immunity in offspring, MMCs prime the expansion of fetal regulatory T (Treg) cells that avert maternal–fetal conflict during pregnancy (16) and confer reproductive fitness in female offspring (17). In contrast to these adaptive T-cell paradigms, maternally derived innate immune cells appear to regulate host defense through antigen-agnostic pathways. MMCs in the fetal bone marrow promote the differentiation of CD11b^+^GR-1^lo/neg^ monocytes that corelate with protection from neonatal CMV infection in mice (18). MMCs are also associated with reduced risk of early life infections in humans, especially in infant boys (18).

MMCs may have sex-differential functions in offspring, contributing to protection against complications in next-generation pregnancies of female offspring via Treg induction (17), while promoting immunity to early life respiratory infection in male offspring via innate immune pathways (18). Offspring female sex is also associated with increased presence of MMCs in infants and young children (19, 20). Distinct from homeostatic cell microchimerism, maternal inflammation during pregnancy is well known to exert sex-differential effects on offspring. Male rat blastocysts are more susceptible to pregnancy-associated inflammation than female blastocysts (21). Maternal immune activation during pregnancy with poly I:C leads to social and behavioral changes specifically in male offspring, driven by transplacental cytokine transfer (22). Whether maternal infection influences MMCs and their function is unknown.

Most infections experienced during human pregnancies are mild and often self-resolve (23). In this study, we leveraged a pregnancy-associated, low-pathogenicity transient infection model to determine the impact of maternal immune activation on MMCs in offspring. Male offspring of previously infected dams harbored more MMCs that promoted the development of type 17 T effector cells and contributed to better health outcomes after infection as young adults. Our studies identify a sex-specific function of MMCs that is revealed upon maternal infection.

## Results

### Pregnancy-associated maternal infection increases MMCs in offspring

**Figure 1:**
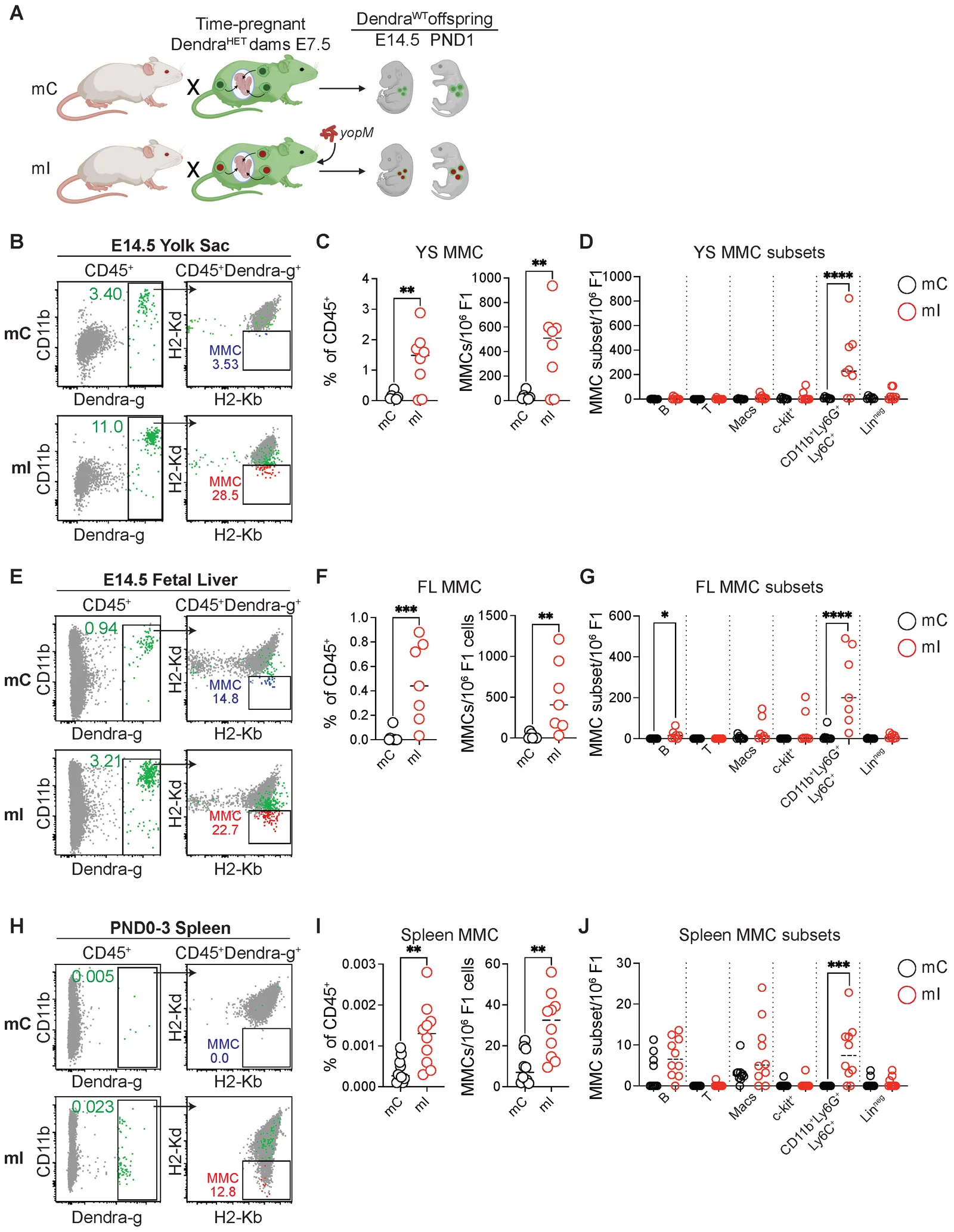
Pregnancy-associated maternal infection increases MMCs in offspring. **(A)** Allogeneic matings of Dendra^HET^ females with BALB/cJ males were set up to generate Dendra^WT^ offspring. Timed-pregnant Dendra^HET^ dams were orally administered *Y. pseudotuberculosis yopM* at embryonic day 7.5 (E7.5). Dendra^WT^ offspring of infected (mI) and control (mC) mothers were analyzed at E14.5 and postnatal day 1 (PND1). **(B)** E14.5 yolk sac (YS): (Left) Representative flow cytometry dot plots showing gating strategy to identify Dendra-g^+^ cells among CD45^+^ cells. Numbers indicate frequency of Dendra-g^+^ cells within CD45^+^ gate. (Right) Dendra-g^+^ cells were further analyzed for expression of H2-Kb and H2-Kd and MMCs were identified as Dendra-g^+^H2-Kb^+^H2-Kd^neg^. Numbers indicate frequency of MMCs within Dendra-g^+^ gate (gray: total CD45^+^ cells; green: Dendra-g^+^ cells; blue: MMCs in mC offspring; red: MMC in mI offspring). **(C)** E14.5 YS: (Left) Frequency of MMCs within CD45^+^ cells. (Right) Numbers of MMCs per million F1 cells (mC *n* = 7, mI *n* = 8). **(D)** E14.5 YS: Subsets of MMCs were identified as CD19^+^ B cells, CD3e^+^ T cells, CD11b^+^F4/80^+^ macrophages (macs), c-kit^+^ cells, CD11b^+^F4/80^neg^Ly6G^+^Ly6C^+^ myeloid cells, and Lin^neg^ (Lin: CD3e, CD19, CD11b, F4/80, Ly6G, Ly6C, and c-kit). Plot shows number of MMCs per million F1 cells (mC *n* = 7, mI *n* = 8). **(E)** E14.5 fetal liver (FL): Gating strategy to identify MMCs as in **(B)**. **(F)** E14.5 FL: Frequency and number of MMCs as in **(C)** (mC *n* = 9, mI *n* = 7). **(G)** E14.5 FL: MMC subsets as in **(D)** (mC *n* = 9, mI *n* = 7). Data in **(B–G)** are representative of two independent experiments using two pregnant dams per group and three to five offspring per pregnancy. **(H)** PND0–3 Spleen: Gating strategy to identify MMCs as in **(B)**. **(I)** PND0–3 Spleen: Frequency and number of MMCs as in **(B)** (mC *n* = 10, mI *n* = 10). **(J)** PND0–3 Spleen: MMC subsets as in **(C)** (mC *n* = 10, mI *n* = 10). Data in **(H–J)** are from five independent experiments using three to five pregnant dams per group and two to five offspring per pregnancy. **(C, F, I)** *t*-test ** < 0.0021, *** <0.0002. **(D, G, J)** One-way ANOVA adjusted *p*-value: *** <0.0002, **** <0.0001. Summary of statistical analyses are shown in **Supplementary Data 1**.

To identify chimeric cells in mouse offspring, we generated female heterozygous PhAM^excised^ mice (Dendra^HET^; fully backcrossed to C57BL/6 strain background) that express a green fluorescent protein called Dendra from a ubiquitous mitochondrial promoter (24). Allogeneic matings, which have previously been shown to increase maternal cell trafficking to offspring (25), of Dendra^HET^ female with Balb/c male mice were set up to generate WT progeny (henceforth called Dendra^WT^ to indicate WT progeny of Dendra-expressing mothers) (**Figure 1A**). Dendra^WT^ progeny (that do not express endogenous Dendra-g protein) were analyzed for the presence of Dendra-g^+^ green-fluorescent maternal cells by flow cytometry. To assess the impact of pregnancy-associated infection on MMCs, we utilized a published animal model of transient, low-pathogenicity intestinal infection with an attenuated *yopM* mutant strain of *Yersinia pseudotuberculosis* (26, 27). Timed-pregnant Dendra^HET^ dams were infected with *yopM* mutant at E7.5 when primitive hematopoiesis is initiated in the yolk sac (YS). *yopM*-infected dams experienced a transient weight loss after infection with peak bacterial shedding in stools at 3 to 4 days post infection (**Supplementary Figures 1A, B**). Maternal *yopM* infection altered splenic distribution of numerous immune subtypes, with a drop in total CD45^+^ hematopoietic cells 5 days after infection compared to uninfected pregnant dams (**Supplementary Figures 1C, D**). Within CD45^+^ cells, there were significant redistribution of immune subtypes with increases in CD11b^+^ myeloid cells including CD11b^+^F4/80^+^ macrophages and CD11b^+^Ly6G^+^Ly6C^+/neg^ cells (**Supplementary Figures 1C, D**).

We first compared the distribution and phenotype of MMCs in the extraembryonic YS and fetal liver (FL) tissues of concepti from naïve (mC) versus infected (mI) dams at E12.5–E14.5. Littermate microchimeric cells that are acquired from adjacent fetuses in multi-embryonic murine pregnancies (28) were excluded from analyses by staining with MHC haplotype antibodies to identify cells of maternal B6 origin (**Figures 1B, E, H**). Maternal infection during pregnancy increased the frequency and numbers of MMCs in both the YS and FL (**Figures 1B–G**). As reported previously (18), MMCs were a heterogeneous mix of CD45^+^ hematopoietic cells including innate and adaptive immune cells (**Figures 1D, G**; **Supplementary Figures 2B–E**). However, maternal infection specifically increased the frequency of CD11b^+^ MMCs in the YS and FL of fetuses (**Figures 1D, G**). The CD11b^+^ cells were primarily F4/80^neg^Ly6G^+^Ly6C^+^, a phenotype that resembled murine granulocytic myeloid-derived suppressor cells (MDSCs) (**Supplementary Figures 2B, D**) (29). We also analyzed newborn mice [postnatal day (PND) 0–3], and while the overall frequency of MMCs in the spleen was significantly lower compared to fetal tissues, there were similar increases in the frequency and numbers of MMCs and CD11b^+^ MMCs in pups delivered by previously infected dams compared to naïve dams (**Figures 1H–J**).

### Increased persistence of MMCs in male offspring of previously infected dams

**Figure 2:**
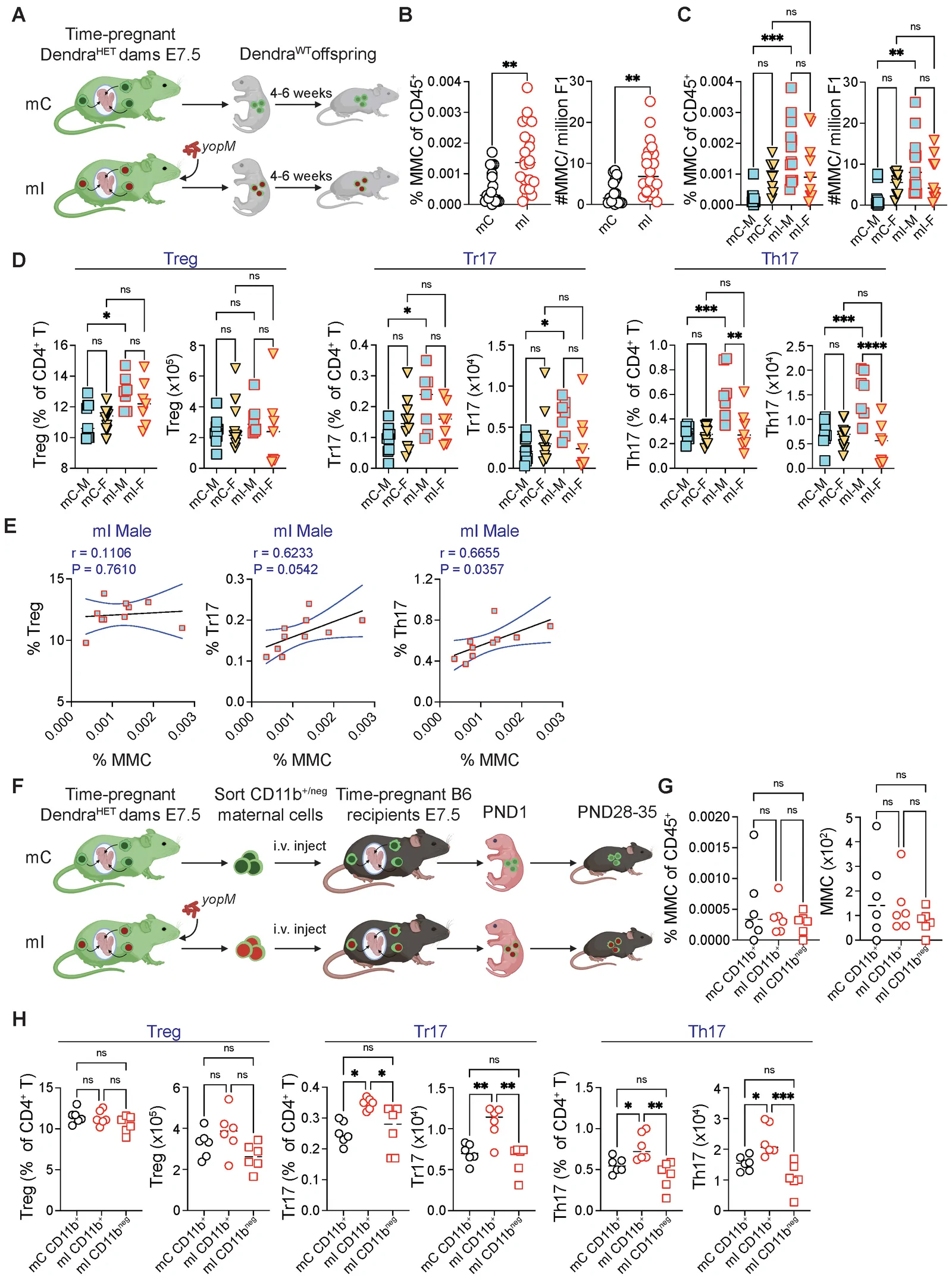
Pregnancy-associated maternal infection increases MMCs in male offspring that correlates with increased splenic CD4^+^ RORγt^+^ T cells. **(A)** Dendra^WT^ offspring of uninfected (mC) or previously infected dams (mI) were analyzed at 4–6 weeks of age. **(B)** Frequency and numbers of MMCs within CD45^+^ cells in spleen (mC *n* = 17, mI *n* = 22; *t*-test *p* ** < 0.0021). **(C)** Frequency and numbers of MMCs within CD45^+^ cells in spleens of male and female mice (mC male *n* = 9, mC female *n* = 8, mI male *n* = 13, mI female *n* = 9; data are from five independent experiments using two to three pregnant dams per group and two to five offspring per pregnancy). **(D)** Frequency and numbers of CD4^+^FOXP3^+^ Treg cells, CD4^+^FOXP3^+^RORγt^+^ Tr17 cells, and CD4^+^RORγt^+^ Th17 cells in spleen (mC male *n* = 9, mC female *n* = 10, mI male *n* = 7, mI female *n* = 7; data are from three independent experiments using two to three pregnant dams per group and two to four offspring per pregnancy). **(E)** Correlation analysis of the percentage of splenic MMCs versus the percentage of Treg, Tr17, and Th17 cells in male mI Dendra^WT^ offspring. The black line represents the linear regression, and the blue line indicates the nonparametric Spearman correlation with a 95% confidence interval. **(F)** Timed-pregnant Dendra^HET^ dams at post-coital day 7.5 were orally administered *Y. pseudotuberculosis yopM*. Five days post-infection, MACS enriched CD11b^+^ and CD11b^neg^ cells from spleens of uninfected (mC) and infected (mI) dams were i.v. injected into timed-pregnant B6 female recipients at post-coital day 7.5. Offspring of recipient B6 dams were analyzed at PND28–35. **(G)** Frequency and numbers of Dendra-g^+^ MMCs within CD45^+^ cells (mC CD11b^+^ *n* = 6, mI CD11b^+^ *n* = 6, mI CD11b^neg^ *n* = 6). **(H)** Frequency and numbers of Treg cells, Tr17 cells, and Th17 cells in spleen (mC CD11b^+^ *n* = 6, mI CD11b^+^ *n* = 6, mI CD11b^neg^ *n* = 6). Data in **(F–H)** are representative of two independent experiments using two pregnant dams per group and four to six offspring per pregnancy. One-way ANOVA adjusted *p*-value: “ns” not significant, * <0.033, ** < 0.0021, *** <0.0002, **** <0.0001. Summary of statistical analyses are shown in **Supplementary Data 1**.

We next assessed whether pregnancy-associated maternal infection altered persistence of MMCs in adult offspring. We analyzed mice after weaning at 4–6 weeks of age (**Figure 2A**) and, consistent with the early time points, observed an increase in frequency and numbers of MMCs in the spleen of adult offspring of previously infected dams (**Figure 2B**). A secondary analysis to address sex as a biological variable revealed that male offspring of previously infected mothers harbored significantly more MMCs compared to male offspring of control mothers (**Figure 2C**), which was confirmed by a two-way analysis of variance (ANOVA) that yielded a significant sex and maternal infection interaction (**Supplementary Figure 3A**) (30). Female offspring maintained stable baseline levels regardless of maternal infection status. A reanalysis of the fetal data segregated by sex, however, did not show significant interaction even though male fetuses appeared to have increased MMCs in the YS and FL during gestation (**Supplementary Figures 3B, C**). These data suggested that postnatal mechanisms likely enforce sex-specific differences in MMC homeostasis.

### Increased MMCs in male offspring of previously infected dams correlates with increased splenic RORγt^+^ T cells

Maternal infection during pregnancy did not alter the distribution of major immune subsets in the spleen of offspring (**Supplementary Figure 4**). An intestinal tissue-specific T helper 17 effector response to postnatal microbial exposure was previously reported in offspring of prenatally infected dams (26). However, we did not observe a significant increase in RORγt-expressing CD4^+^ T (Th17) cells in the small intestine of 4- to 5-week-old offspring (**Supplementary Figure 5A**). The genetic background of animals influences gut microbial diversity (31), which likely has an impact on Th17 cell homeostasis. The mixed B6-Balb/c background of offspring of allogeneic matings as well as the earlier timing of maternal infection at E7.5 in our model may contribute to the lack of a gut-specific Th17 response as seen previously. However, maternal infection was indeed associated with a significant increase in RORγt^+^ and IL-17^+^ Th17 cells in the spleen of male offspring compared to those from uninfected dams (**Figure 2D**; **Supplementary Figures 5B–D**). There were no differences in IFN-γ and interleukin-4 (IL-4) producing Th1 and Th2 cells, respectively (**Supplementary Figures 5B–D**). There was also a significant positive correlation between MMCs and Th17 cells in the spleen of male, but not female offspring (**Figure 2E**; **Supplementary Figure 5E**). While distribution of CD4^+^FOXP3^+^ Treg cells was unaffected, the frequency of RORγt expressing Treg cells (Tr17), which are induced by microbes in the gut as well as by immunization in peripheral lymph nodes (32–34), was also increased and correlated with frequency of MMCs (**Figures 2D, E**). There were no changes in other RORγt expressing cell subsets including TCRb^neg^CD3^neg^RORγt^+^ cells, iNKT17 cells, and CD11c^+^RORγt^+^ dendritic cells (**Supplementary Figures 5B, F**). Thus, transient pregnancy-associated maternal infection influenced the homeostasis of RORγt-expressing CD4^+^ T cells in offspring that correlated with the frequency of MMCs.

Maternal IL-6 produced during pregnancy-associated infection can induce intestinal Th17 responses in offspring (26). To exclude the contribution of trans-placentally transferred soluble factors including cytokines and antibodies, and to specifically determine if maternal cells regulated Th17 cell homeostasis in offspring lymphoid tissues, we conducted an adoptive transfer experiment (**Figure 2F**). We focused on CD11b^+^ MMCs as their frequency was increased in male offspring of infected dams at the fetal and immediate postnatal stages (**Figure 1**). Sorted Dendra-g^+^ CD11b^+^ and CD11b^neg^ splenocytes from Dendra^HET^ pregnant females 5 days after *yopM* infection were intravenously injected into timed-pregnant C57BL/6 females at E7.5. Dendra-g^+^CD11b^+^ cells sorted from uninfected pregnant females were used as controls. There were no differences in the frequency of transferred maternal cells acquired by offspring across groups (**Figure 2G**). However, offspring of B6 females that received CD11b^+^ cells from previously infected mothers showed elevated Tr17 and Th17 cells in the spleen compared to offspring of recipients of CD11b^+^ cells from control uninfected mothers (**Figure 2H**). The ability to induce RORγt^+^ T-cell responses was restricted to the CD11b^+^ population as offspring of recipients of CD11b^neg^ cells from previously infected mothers were unable to do so. Furthermore, the effect of acquired CD11b^+^ cells was specific to RORγt^+^ T cells as the frequency of Treg cells remained unchanged. These data suggest that transient maternal infection during pregnancy enhanced the ability of MMCs to promote a type 17 T effector cell response in the offspring.

### Maternal infection increases resistance of male offspring to *Salmonella* typhimurium infection

**Figure 3:**
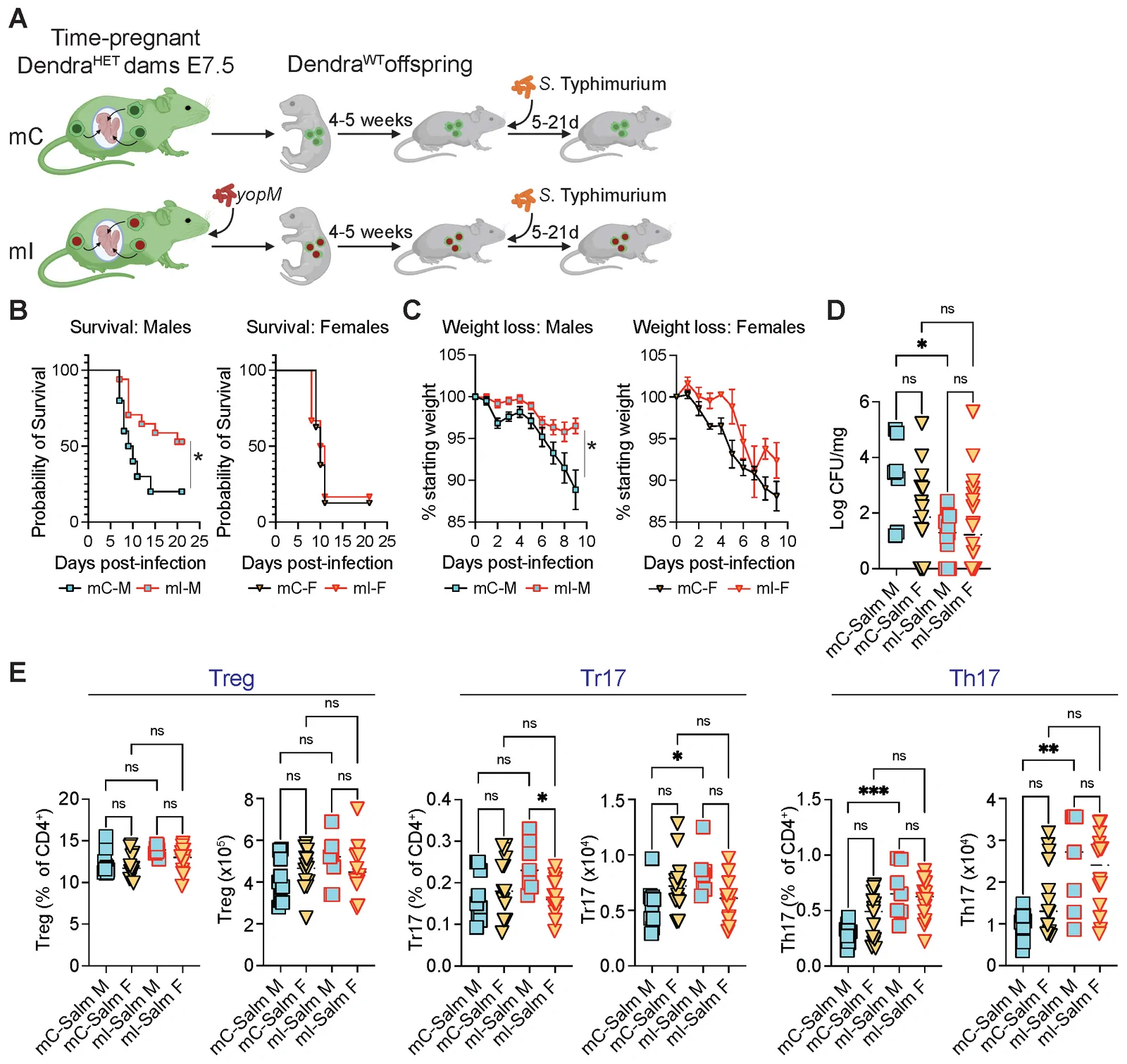
Pregnancy-associated maternal infection alters susceptibility of male offspring to *Salmonella* infection. **(A)** Dendra^WT^ offspring of uninfected (mC) or previously infected dams (mI) were orally administered *Salmonella* Typhimurium (SL1344 mutant) at PND28–35. **(B)** Survival curve of male and female offspring after *Salmonella* infection (mC-Salm M *n* = 10, mI-Salm M *n* = 17, mC-Salm F *n* = 8, mI-Salm F *n* = 6; *p* * <0.03). **(C)** Percent weight change after *Salmonella* infection (mC-Salm M *n* = 9, mI-Salm M *n* = 17, mC-Salm F *n* = 6, mI-Salm F *n* = 5; *p* * <0.03). **(D)** Splenic bacterial burden 5 days after *Salmonella* infection (mC-Salm M *n* = 7, mC-Salm F *n* = 15, mI-Salm M *n* = 12, mI-Salm F *n* = 18). **(E)** Frequency and numbers of Treg cells, Tr17 cells, and Th17 cells in spleen (mC-Salm M *n* = 13, mC-Salm F *n* = 17, mI-Salm M *n* = 7, mI-Salm F *n* = 14). Data in **(B–E)** are from two to four independent experiments using three to four pregnant dams per group and four to six offspring per pregnancy. One-way ANOVA adjusted *p*: “ns” not significant, * <0.033, ** < 0.0021, *** < 0.0002, **** <0.0001. Summary of statistical analyses are shown in **Supplementary Data 1**.

We next sought to determine if there were functional consequences of the association between MMCs from previously infected mothers and the male offspring’s own Th17 response. IL-17-producing RORγt^+^ cells have been shown to be protective in *Salmonella* infection (35). We, therefore, challenged male and female offspring of previously infected and control dams with *Salmonella* Typhimurium at PND25–30 (**Figure 3A**). Male offspring of previously infected dams had significantly improved survival following acute *Salmonella* infection than the male offspring of uninfected dams (**Figure 3B**). They lost significantly less weight and displayed a small but statistically significant decrease in splenic pathogen burdens (**Figures 3C, D**; **Supplementary Figure 6**). These differences in handling *Salmonella* were associated with significantly higher Tr17 and Th17 cells, but not Treg cells, in the male offspring of infected mothers (**Figure 3E**).

### Depletion of CD11b^+^ MMCs alters Th17 cell homeostasis and susceptibility to infection in male offspring

**Figure 4:**
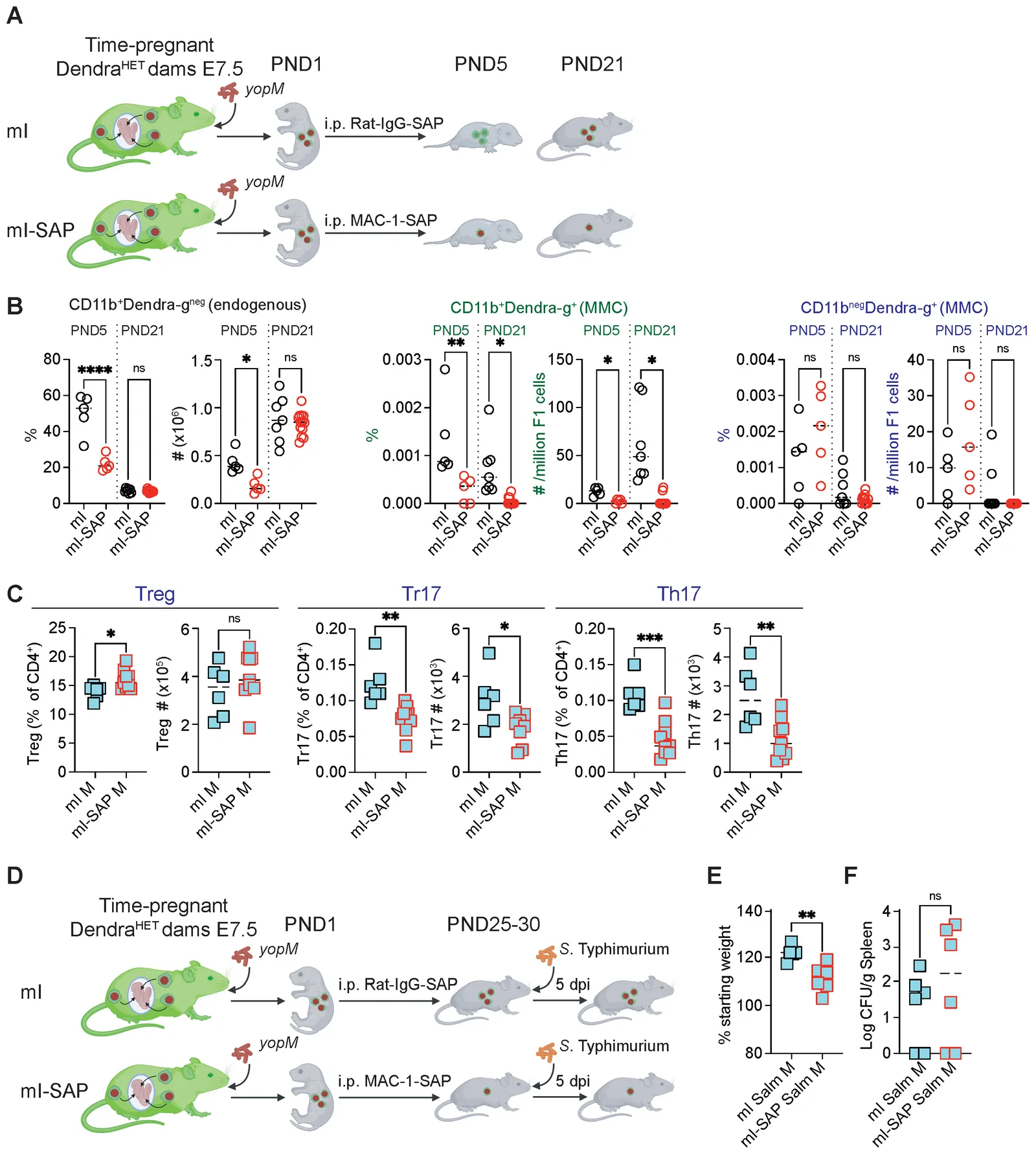
Loss of CD11b^+^ MMCs alters type 17 T effector cell profile and susceptibility to *Salmonella* infection in male offspring. **(A)** Newborn male (PND0) Dendra^WT^ offspring of previously infected dams (mI) were injected with control Rat-IgG SAP (mI Ctrl) or MAC1-SAP (mI SAP) antibody to deplete CD11b^+^ cells. Pups at PND5 and PND21 were analyzed for the presence of MMCs and distribution of effector T cell subsets. **(B)** Frequency and numbers of (left) endogenous (offspring origin) Dendra^neg^CD11b^+^ cells, (middle) CD11b^+^ MMCs, and (right) CD11b^neg^ MMCs in PND5 and PND21 offspring of previously infected dams that were treated with either control rat-IgG-SAP (mI) or Mac-1-SAP (mI-SAP) antibody (PND5: mI *n* = 5, mI-SAP *n* = 5; PND21: mI *n* = 7, mI-SAP *n* = 13). **(C)** Frequency and numbers of splenic Treg cells, Tr17 cells, and Th17 cells in control and Mac-1-SAP-treated male PND28–35 offspring of previously infected dams (mI M *n* = 6, mI-SAP M *n* = 9). Data in **(B, C)** are from two to four independent experiments using offspring of three to four infected dams in each group. One-way ANOVA adjusted *p*: “ns” not significant, * <0.033, ** < 0.0021, *** < 0.0002, **** <0.0001. **(D)** Newborn male (PND0) Dendra^WT^ offspring of previously infected dams (mI) were injected with control Rat-IgG SAP (mI Ctrl) or MAC1-SAP (mI SAP) antibody to deplete CD11b^+^ cells. Mice were infected with *Salmonella* at PND 25–30 and analyzed 5 days post infection. **(E)** Percent change in body weight 5 days after *Salmonella* infection in male offspring (mI Salm M *n* = 6, mI-SAP Salm M *n* = 6). **(F)** Splenic bacterial burden 5 days after *Salmonella* infection (mI Salm M *n* = 6, mI-SAP Salm M *n* = 6). Data in **(E, F)** are from two independent experiments using offspring of three to four infected dams in each group. *t*-test *p*: “ns” not significant, * <0.033, ** < 0.0021, *** < 0.0002, **** <0.0001. Summary of statistical analyses are shown in **Supplementary Data 1**.

Data so far pointed to a role for maternally conditioned CD11b^+^ MMCs in regulating type 17 T-cell responses in male offspring. To determine whether CD11b^+^ MMCs are required for this phenomenon, we depleted MMCs in newborn male mice using a saporin-conjugated antibody to Mac-1 (CD11b) (36). Male offspring of previously infected dams were administered either Mac-1-SAP or rat-IgG-SAP antibody on the day of birth (PND0) and analyzed on PND5 and PND21 after treatment (**Figure 4A**). Mac-1-SAP treatment is expected to kill all CD11b^+^ cells in newborn mice, including CD11b-expressing maternal cells and the offspring’s own CD11b-expressing myeloid cells. However, we reasoned that while gestationally transferred MMCs would be irreversibly killed, the offspring’s own CD11b^+^ compartment would be replenished from new precursors arising in the bone marrow. Endogenous CD11b^+^ cells were indeed significantly reduced 5 days after Mac-1-SAP treatment, but their numbers recovered by PND21 (**Figure 4B**). CD11b^+^ MMCs were also reduced at 5 days post treatment but, unlike endogenous CD11b^+^ cells, remained significantly reduced at PND21 (**Figure 4B**). There were no differences in CD11b^neg^ MMCs at either time point (**Figure 4B**). The decrease in CD11b^+^ MMCs was accompanied by a significant decrease in Tr17 and Th17 cells at PND21 in Mac1-SAP-treated male offspring of previously infected dams (**Figure 4C**). Finally, we challenged Mac-1-SAP- and control rat-IgG-SAP-treated offspring with *Salmonella* Typhimurium at PND25–30 (**Figure 4D**). Despite splenic bacterial burden not being significantly different at day 5 after infection, Mac-1-SAP-treated male offspring of previously infected dams appeared to do more poorly and exhibited greater weight loss compared to control rat-IgG-SAP-treated male offspring after *Salmonella* infection (**Figures 4E, F**).

## Discussion

Vertically transferred maternal factors play crucial roles in regulating offspring immunity in early life. Here, we showed that pregnancy-associated transient infection drives a significant accumulation of MMCs early in gestation within embryonic hematopoietic niches, specifically the YS and FL at E14.5. Increased MMCs specifically in male offspring of previously infected mothers promoted a RORγt-expressing effector T-cell (type 17) response that decreased susceptibility to infection. These findings highlight a sex-differential function of MMCs that is revealed upon maternal infection during pregnancy.

Allogeneic MMCs induce Treg cells in female offspring to establish tolerance to non-inherited maternal antigens (NIMAs) and enable their persistence into adulthood. In our studies, we consistently observed increased frequency of MMCs in young-adult female offspring of naïve mothers compared to male offspring, suggesting distinct sex-specific mechanisms that maintain MMCs in the long term. Maternal infection during pregnancy, however, altered this dynamic and led to an induction of MMCs in male offspring of infected mothers compared to those from naïve mothers. Maternal cells in male offspring induced a pro-inflammatory Tr17/Th17 program in lymphoid tissues that conferred protection from severe *Salmonella* infection in young adulthood. The mechanisms by which MMCs induce type 17 responses is yet unknown. Given the phenotypic resemblance of MMCs to CD11b^+^Ly6G^+^Ly6C^+^ MDSCs, possible mechanisms of action could involve production of arginase-1 and reactive oxygen species that have been shown to regulate Th17 differentiation in models of systemic lupus erythematosus and lupus nephritis (37, 38). Of note, there was a significantly greater impact on overall health and body weight than on control of bacterial load in infected offspring. Disease tolerance is a phenomenon whereby the host prioritizes tissue damage control over elimination of pathogen as a means to preserve long-term homeostasis (39). It is intriguing to speculate that MMCs safeguard their own survival and the reproductive fitness of their female host by inducing NIMA-specific Treg cells but may influence the state of disease tolerance and infection risk in their male hosts by inducing a type 17 T-cell response. This remains a hypothetical model based on our observations combined with previously published paradigms of NIMA tolerance and will need to be confirmed in future experiments. Sex differences in immunity lead to enhanced vaccine responses, greater resistance to infection, and an increased risk of autoimmune conditions in females relative to males (40). Newborn males are particularly vulnerable to infections and death compared to females (41). However, increased MMCs in male offspring correlates with reduced early life infections in humans (18). Our findings extend this observation to include a role for the maternal immunological experience that influences acquired MMCs to provide immune benefits to male offspring in infectious settings.

Sex-specific differences in tissue immunity have been reported. For example, type 2 immunity is lower in male mice compared to female mice after allergen exposure in a model of allergic airway inflammation (42). It is noteworthy that we observed an impact of MMCs on male offspring T effector phenotype specifically in the spleen but not at tissue sites such as the small intestine. While MMCs have been reported in multiple tissues (4), they appear to be enriched at sites of hematopoiesis including the fetal bone marrow (18) and, in our studies, the YS and FL. Furthermore, in contrast to their presence in the female uterus, they are notably absent in male reproductive tissues of the prostrate (17). MMCs may thus selectively take up residence in specific tissues where they serve as an alloantigen source to influence T-cell immunity. We also observed that sex differences in MMC numbers only manifested during the postnatal period and were absent during fetal development. One possibility is that fluctuations in sex-specific hormone levels influenced by age and external environmental factors such as the microbiota may differentially regulate the homeostasis of maternal cells in male and female offspring (43, 44). Androgens and androgen receptor signaling were recently shown to regulate skin T-cell expansion in a sex-specific manner (45). This sex-differential persistence may be mechanistically driven by postnatal fluctuations in sex steroids, as circulating myeloid lineages express functional androgen and estrogen receptors (46, 47). Specifically, androgen signaling has been shown to actively promote the expansion and suppressive function of granulocytic MDSCs (48), which match the phenotype of MMCs in our model. Future studies should uncover the role of sex hormones in regulating MMC homeostasis.

Infection-induced maternal immune activation is associated with increased expression and trans-placental transport of inflammatory cytokines that influence immune functionality and offspring disease susceptibility. Vertically transferred maternal IL-6 is one conveyor of maternal inflammation to the developing fetus, affecting functional brain networks in the immediate postnatal period (49). Maternal IL-6 also imprints altered functionality into the developing fetal intestine that influences immune responses at barrier surfaces in adulthood (26). In our studies, we showed that the transfer of CD11b^+^ cells from infected mothers into naïve pregnant recipients and their subsequent acquisition by offspring was sufficient to induce the type 17 program in offspring lymphoid tissue. While the adoptive transfer experiments aimed to isolate the cellular contribution of MMCs from the systemic inflammatory environment of an infected dam, the transferred cells themselves could secrete cytokines such as IL-6 that could regulate the type 17 effector phenotype. Future studies will need to address whether maternal infection alters the acquisition and/or persistence of MMCs in offspring and how it imprints distinct functionality in subsets of MMCs, including the CD11b^+^ MMCs, which induces the type 17 T-cell effector program in lymphoid tissues of male offspring.

### Limitations of the study

In proof-of-concept studies, we tracked MMCs in animal models and uncovered their sex differential functions in offspring following transient maternal infection with *yopM*. Maternal immune activation following infection during pregnancy can take different forms depending on the type and severity of infection as well as the involvement of the placenta that may differentially influence acquired maternal cells and their function. Studies using additional challenges combined with consideration of timing of maternal infection as well as maternal parity are warranted to clarify their impact on MMCs. All mice used in our experiments were on the B6 or B6 × Balb/c background and so carried the Nramp1 nonfunctional allele that confers susceptibility to *Salmonella* infection. B6 mice are widely used in studies of salmonellosis and have provided several important insights into pathogenesis and the anti-*Salmonella* immune response. Nevertheless, we acknowledge that our findings must be interpreted with the understanding that they may have been influenced by Nramp1 functionality.

Our study focused on trans-placentally transferred maternal cells and the impact of their depletion on offspring susceptibility to infectious challenge. While our experiments demonstrate that postnatal persistence of CD11b^+^ MMCs is required to maintain the type 17 program, it does not preclude parallel prenatal developmental programming. MMCs are also transferred through breastmilk in early life, and we might have missed defining a role for these cells, particularly in regulating mucosal immunity in offspring. MMCs are a rare population of cells, and their study in humans has been limited by available methods to identify them and establish causal relationships in disease settings. We acknowledge that developmental timelines are distinct in humans and mice, and while it may be premature to draw direct parallels between our findings and observations made in humans, our studies clearly call for dedicated exploration of maternal immune challenges and their impact on MMCs.

### Mice

Animal studies were conducted under protocols approved by the Institutional Animal Care and Use Committee of Massachusetts General Hospital. The following animal lines were used: PhAM^excised^ mice [B6;129S-Gt(ROSA)26Sor^tm1.1(CAG-COX8A/Dendra2)Dcc^/J; Jax strain 018397], C57BL/6 mice (Jax strain: 00664), and BALB/cJ mice (Jax strain 000651). PhAM^excised^ (Dendra^HOM^) mice were mated with C57BL/6 mice to generate Dendra^HET^ females that were used as breeders in allogeneic matings with Balb/cJ mice. Mice were housed in HPPF (*Heliobacter-* and *Pasteurella pneumotropica-*free) facilities before mating and transferred to BL-2 facilities for infection. Sex and age of all mice used are reported in figure legends. The *Y. pseudotuberculosis yopM* strain was a gift from Dr. Igor Brodsky and Dr. James Bliska (27). The SL1344 strain of *Salmonella* Typhimurium was a gift from Dr. Beth McCormick.

### Timed matings

Littermate female Dendra^HET^ mice from a Dendra^HOM^ and C57BL/6 cross were used in all experiments. Singly housed experienced Balb/cJ male mice were mated with Dendra^HET^ females that were in proestrus or estrus. Presence of vaginal plug was monitored over the next 3 to 4 days, and males were separated from the breeding cage once a plug was observed. The morning on which plugs were observed was designated as embryonic day 0.5. Offspring were weaned at 3 weeks after birth and age-matched male and female mice were used in experiments as specified.

### Yersinia pseudotuberculosis infection

The *yopM* mutant strain of *Y. pseudotuberculosis* 32777 was grown in 2 mL of YT Broth (2×) (Sigma) at 30 °C with shaking at 250 rpm. Overnight culture (100 µL) was diluted 10-fold with phosphate-buffered saline (PBS), and the OD value was obtained with a spectrophotometer absorbance reading at 600 nm. Overnight cultures were then appropriately diluted with PBS to reach an inoculum size of ~ 2 × 10^7^ colony-forming units (CFUs)/100 µL. Timed-pregnant female mice were administered 100 µL of bacterial suspension by oral gavage. Stool samples collected from infected pregnant dams were weighed, and fecal DNA was extracted using the QIAmp Fast DNA Stool Mini Kit (Qiagen). The *yscF* gene was quantified against a standard curve by quantitative polymerase chain reaction (PCR) (forward primer 5′-ATGAGTAACTTCTCTG-GATTTACG-3′, reverse primer 5′-TTATGG-GAACTTCTGTAGGATG-3′). Quantitative PCR was performed using the QuantStudio 3 Real-Tine PCR system with a well volume of 20 µL.

### *Salmonella* typhimurium infection

The SL1344 strain of *S.* Typhimurium was cultured in LB with 50 µg/mL streptomycin at 37 °C while shaking at 250 rpm. After 8 h, 10 µL of the shaking culture was back diluted into 10 mL of fresh LB with 50 µg/mL streptomycin and incubated overnight without shaking. Overnight standing cultures were centrifuged and diluted to 2 × 10^7^ CFU per 100 µL in PBS for infection. Offspring (28–35 days old) of Dendra^HET^ × Balb/cJ matings were infected by oral gavage. At 5 days after infection, harvested tissues were weighed and homogenized in 0.5% Triton in PBS. Serial dilutions were spread plated on LB agar and incubated overnight at 37 °C before calculating CFUs.

### Tissue collection and single-cell preparation

Single-cell suspensions were prepared from the spleens of newborn and adult animals following mechanical homogenization as described previously (50). Red blood cells (RBCs) were lysed using ACK lysis buffer. Cells from the small intestine lamina propria were isolated after removing epithelial fraction followed by digestion in Liberase (Roche). Embryonic tissues (FL and YS) were homogenized between frosted glass slides and digested in Liberase TL (thermolysin low) as previously described (51). Cells isolated from all tissues were counted on a Countess 3 (Thermo Fisher Scientific) instrument and resuspended in FACS buffer in preparation for staining.

### *In vitro* cell stimulation

Splenocytes were cultured with 1× protein transport inhibitor cocktail (Thermo Fisher Scientific) and 1× cell stimulation cocktail (Thermo Fisher Scientific) for 16 h. Cultures were harvested and cells were stained for surface markers. Cells were then fixed and permeabilized with BD Cytofix/Cytoperm buffer and stained with cytokine antibodies before analysis on a flow cytometry.

### Flow cytometry

Antibody and reagent information is provided in **Supplementary Table 1**. A maximum of 3 × 10^6^ cells were stained with fluorophore-conjugated antibodies. After surface staining, cells were fixed and permeabilized using the Foxp3/Transcription Factor Staining Buffer Set (Thermo Fisher Scientific) and stained with intracellular transcription factor antibodies. Dead cells were excluded using the LIVE/DEAD Fixable Blue Dead Cell Stain Kit (Invitrogen Life Technologies). Samples were acquired on a 5-laser Cytek Aurora spectral analyzer. Single color controls were made using UltraComp eBeads for spectral unmixing on the cytometer.

Flow cytometry-based genotyping was performed to identify Dendra^WT^ and Dendra^HET^ samples for embryonic and newborn time points. Single-cell suspensions from the fetal liver were acquired on the Cytek in plate mode, and Dendra^HET^ samples, in which >98% of total events are Dendra-g^+^, were identified and discarded. Only stained Dendra^WT^ samples were acquired, and the “sample recovery” function and a 30-s water wash between each sample were programmed into the SpectroFlo software that minimized carryover (52). Single-cell suspensions from entire organs at the fetal and postnatal day 1 time points were run through the cytometer. For adult tissues, up to 1 × 10^6^ cells were acquired at a flow rate between 50 and 70 μL/min. The data were analyzed using FlowJo v10 software (BD).

### *In vivo* manipulations

A total of 2 × 10^4^ to 5 × 10^4^ MACS enriched CD11b^+^ and CD11b^neg^ cells (Miltenyi Biotec) from maternal spleens were i.v. injected into recipient females. A single dose of Mac-1-SAP or Rat-IgG-SAP (ATS Bio; 4 μg per animal) was i.p. injected into newborn mice.

### Statistical analysis

GraphPad Prism (v.10.4.1) was used to perform data analyses. Data from all statistical analyses performed are shown in **Supplementary Data 1**. Two-tailed unpaired *t*-tests were used for comparisons between groups at similar time points. Comparisons between male and female offspring of infected and control dams were performed using o-way ANOVA with Tukey’s multiple comparisons test with a single pooled variance. All sex differences were established using two-way ANOVAs combined with Tukey’s multiple comparison test. No randomization was performed, and investigators were not blinded to group allocations.

## Data Availability

The original contributions presented in the study are included in the article/**Supplementary Material**. Further inquiries can be directed to the corresponding author.
