# Supplementary material for: Prenatal maternal infection promotes maternal microchimeric cells to alter infection risk in male offspring
Source: Front Immunol. 2026 Sep 3;17:1869048. doi: 10.3389/fimmu.2026.1869048 (PMC13582634; doi:10.3389/fimmu.2026.1869048)
Supplement: Supplementary file 1 [file SupplementaryFile1.pdf]

Supplementary Materials

Prenatal maternal infection promotes maternal microchimeric cells to alter infection risk in male offspring

Jimena et al

Corresponding author: Nitya Jain, njain@mgh.harvard.edu

The PDF file includes:

Supp Figs 1-6

Other Supplementary material includes:

Supplemental Data 1

Summary of statistical analyses used in manuscript, related to Figures 1-4.

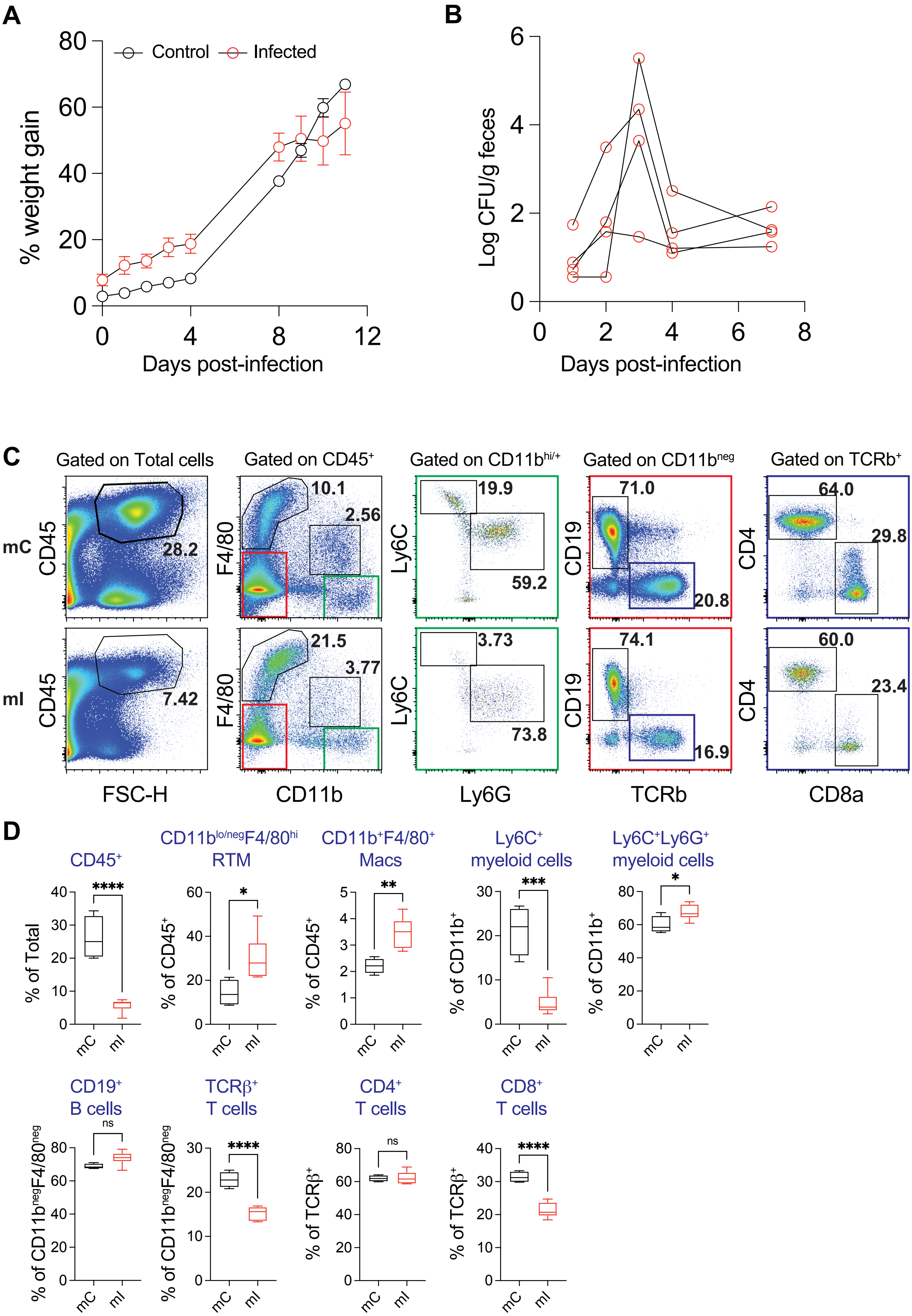

Supplemental Figure 1: ***yopM*** **model of maternally restricted infection**

Supplemental Figure 1: *yopM* models of maternally restricted transient infection, related to Figure 1

Timed-pregnant Dendra^{HET} dams were orally administered *Y. pseudotuberculosis yopM* at embryonic day 7.5 (E7.5).

(**A**) Body weight of control (mC) and *yopM*-infected (mI) dams over the course of pregnancy. (mC n=3; mI n=5)

(**B**) yopM burden in feces at 1, 2, 3, 4 and 7 days after infection (n=5).

(**C**) Maternal splenic immune cell distribution 5 days after *yopM* infection. Representative flow cytometry dot plots show gating strategy to identify immune subsets in mC and mI females.

(**D**) Frequency of CD45^{+} cells, resident tissue macrophages (RTMs; CD11b$^{lo/neg}$F4/80^{hi}), macrophages (Macs; CD11b^{+}F4/80^{+}), CD11b^{+}Ly6C^{+} myeloid cells, CD11b^{+}Ly6C^{+}Ly6G^{+} myeloid cells, CD19^{+} B cells, TCRb^{+} T cells, CD4^{+}T cells and CD8^{+} T cells. (mC n=4, mI n=6; data are representative of 2 independent experiments).

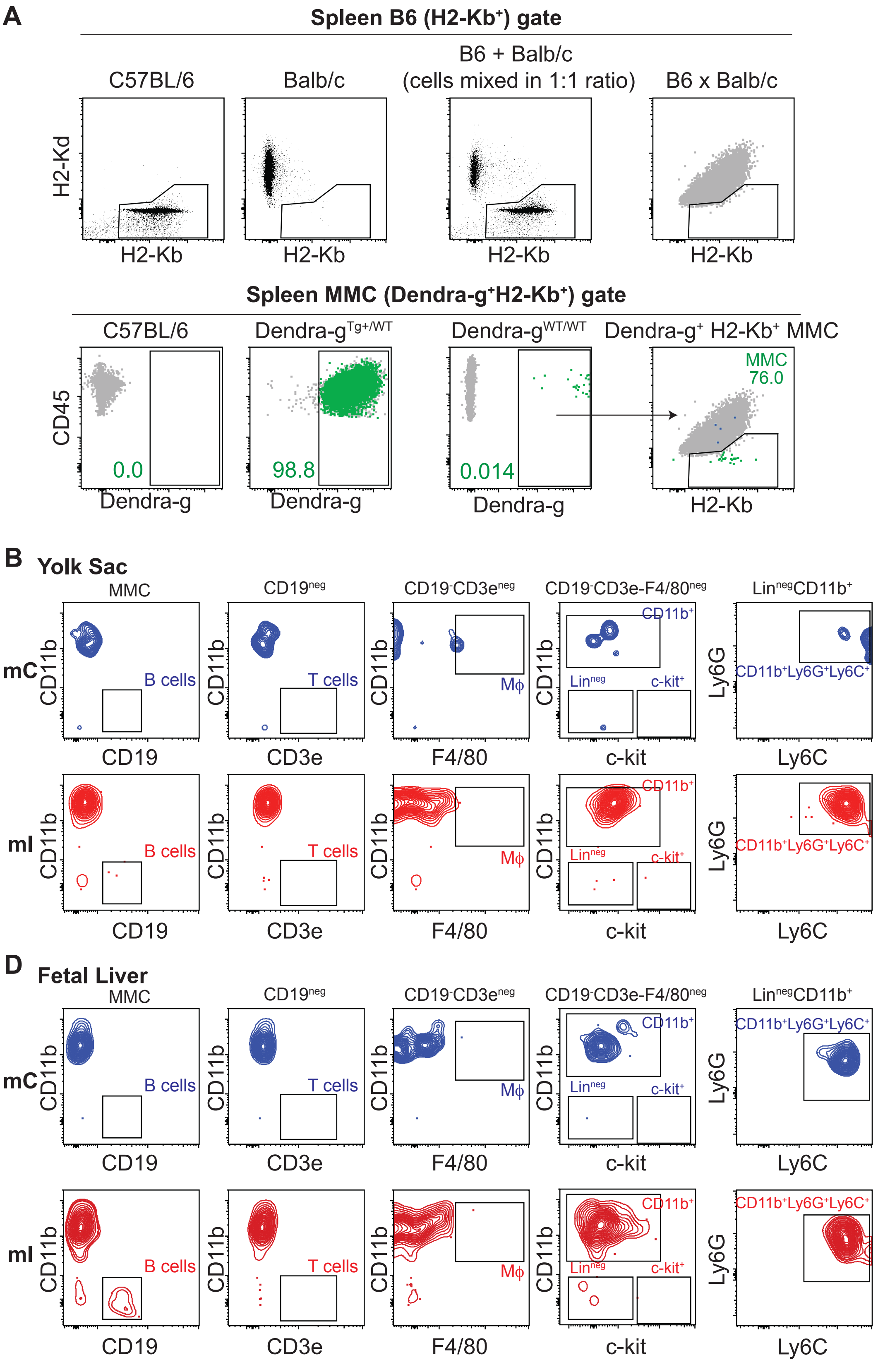

Supplemental Figure 2: Gating strategy to identify MMCs and MMC subsets

Supplemental Figure 2: Gating strategy to identify MMC subsets, related to Figure 1.

(A) MMC gating strategy: (*Top row*) Splenocytes from C57BL/6, Balb/c, and offspring of C57BL/6 and Balb/c mice (B6 X Balb/c) were stained with H2-Kb and H2-Kd antibodies to identify B6 and Balb/c haplotypes. A 1:1 mix of stained B6 and Balb/c cells was also acquired on the cytometer. (*Bottom row*) Splenocytes from C57BL/6, Dendra-g$^{Tg+/-}$ and Dendra$^{WT/WT}$ offspring of a Balb/c male mated with Dendra-g$^{Tg+/-}$ female were analyzed by flow cytometry to set Dendra-g^{+} gate. Dendra-g^{+} cells were further analyzed for H2-Kb an H2-Kd expression to identify MMCs of maternal (B6) origin.

(B-D) Allogeneic matings of Dendra^{HET} females with BALB/cJ males were set up to generate Dendra^{WT} offspring. Timed-pregnant Dendra^{HET} dams were orally administered *Y. pseudotuberculosis yopM* at embryonic day 7.5 (E7.5). Yolk sac (YS) and fetal liver (FL) tissues from E14.5 fetuses from infected (mI) and control (mC) mothers were analyzed by flow cytometry.

(B) Identification of MMCs in YS, FL: (*Left*) Representative dot plots show expression of Dendra-g (x-axis) and CD11b (y-axis) on CD45^{+} cells; green: Dendra-g^{+} cells, gray: Dendra-g^{neg} cells. (*Right*) Dendra-g^{+} cells were further analyzed for expression of MHC haplotypes H2-Kd (Balb/c) and H2-Kb (B6). MMCs of maternal B6 origin were identified as H2-Kd^{neg}H2-Kb^{+}.

(**C**) **Subsets of MMCs in YS**: Dendra-g^{+} cells of maternal B6 origin (H2-Kd^{neg}H2-Kb^{+}) in YS were analyzed for expression of lineage markers to identify CD19^{+} B cells, CD3e^{+} T cells, CD11b^{+}F4/80^{+} macrophages and c-kit^{+} cells. CD11b^{+}F4/80^{neg} cells were analyzed further for expression of Ly6G and Ly6C to identify CD11b^{+}Ly6C^{+}Ly6G^{neg} cells.

(**D**) Frequency of MMC subsets in mC and mI E14.5 YS (mC: n=7, mI: n=8). Error bars are SEM.

(E) Subsets of MMCs in FL: Dendra-g^{+} cells of maternal B6 origin (H2-Kd^{neg}H2-Kb^{+}) in FL were analyzed as in (**C**).

(**F**) Frequency of MMC subsets in mC and mI E14.5 FL (mC: n=9, mI: n=7). Error bars are SEM.

Data in B-F are representative of 2 independent experiments.

A PND28-35 Spleen

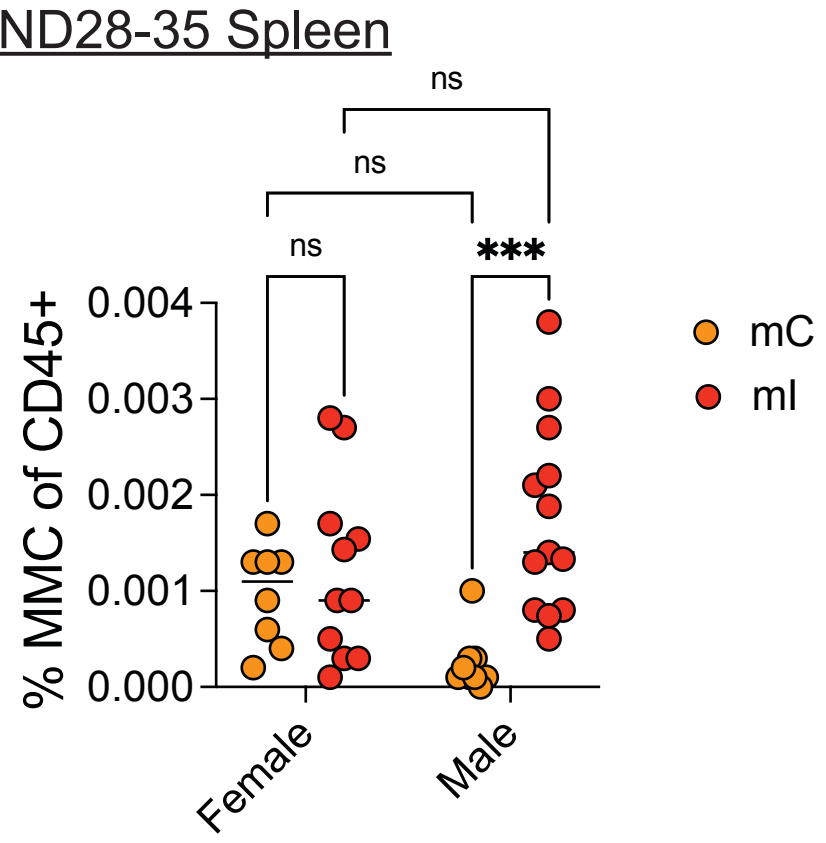

Two-way ANOVA	P value summary
Interaction	*
Offspring sex	ns
Maternal infection	**

B E14.5 YS

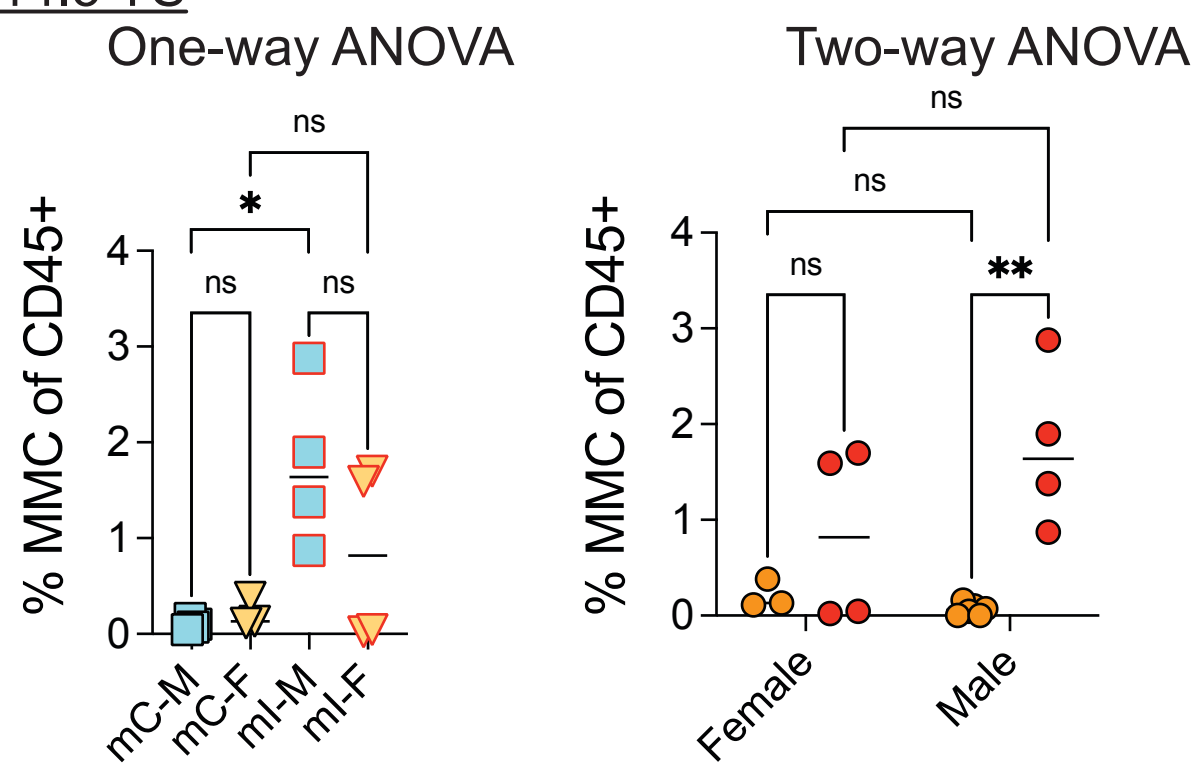

Two-way ANOVA	P value summary
Interaction	ns
Offspring sex	ns
Maternal infection	**

C E14.5 FL

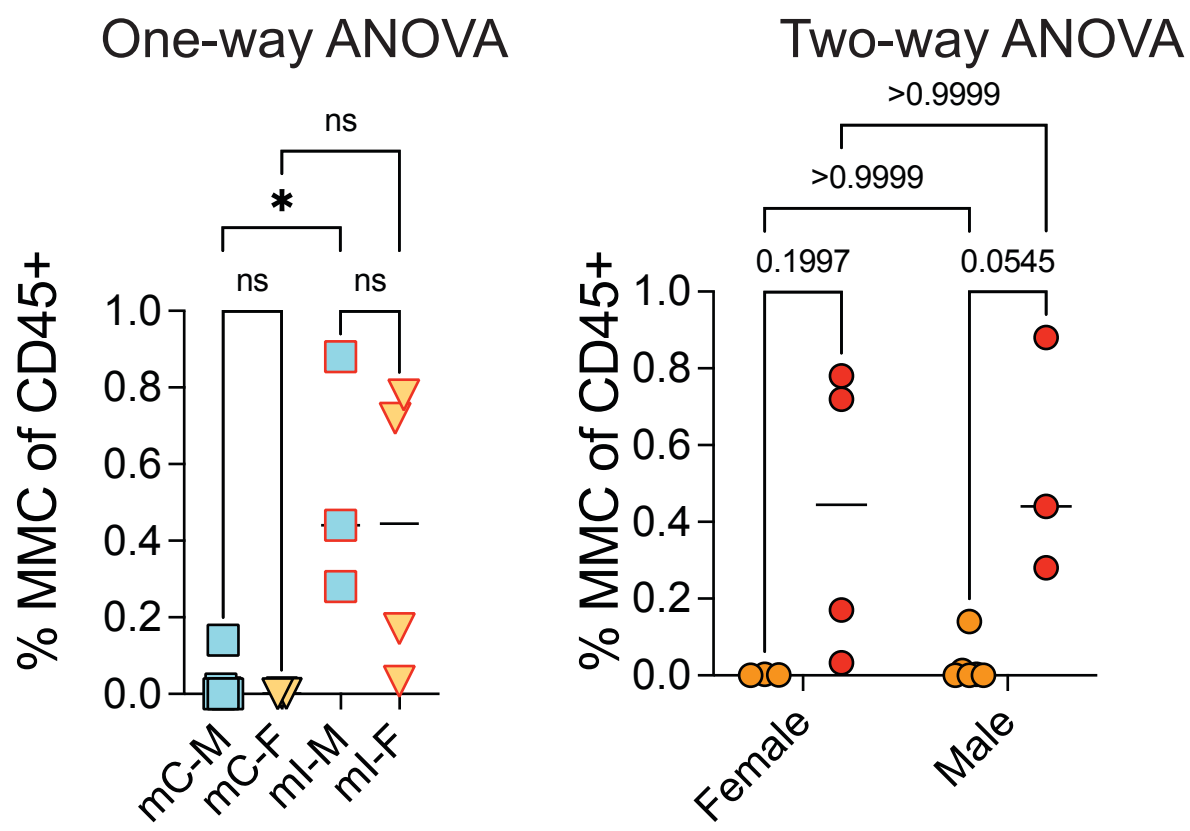

Two-way ANOVA	P value summary
Interaction	ns
Offspring sex	ns
Maternal infection	**

Supplemental Figure 3: Pregnancy-associated maternal infection increases MMCs in male offspring.

Supplemental Figure 3: Pregnancy-associated maternal infection increases acquisition of MMCs in male fetuses, related to Figure 1

Allogeneic matings of $Dendra^{HET}$ females with BALB/cJ males were set up to generate $Dendra^{WT}$ offspring. Timed-pregnant $Dendra^{HET}$ dams were orally administered *Y. pseudotuberculosis yopM* at embryonic day 7.5 (E7.5). Male and female $Dendra^{WT}$ offspring of infected (mI) and control (mC) mothers were analyzed at PND28-35 and E14.5.

(A) Frequency of MMCs within $CD45^{+}$ cells in offspring spleens of previously infected dams segregated by sex. Two-way ANOVA results are shown on the right. (mC male n=9, mC female n=8, mI male n=13, mI female n=9; data are from 5 independent experiments using 2-3 pregnant mice dams per group and 2-5 offspring per pregnancy).

(**B)** E14.5 Yolk Sac (YS): (*Left*) Frequency of MMCs within $CD45^{+}$ cells. One-way ANOVA analysis is shown. (*Right*) Frequency of MMCs within $CD45^{+}$ cells in offspring spleens of previously infected dams segregated by sex. Two-way ANOVA analysis is shown. (mC M n=4, mC F n=3, mI M n=4, mI F n=4; data are from 2 independent experiments using 2 pregnant mice dams per group and 3-5 offspring per pregnancy).

(**C)** E14.5 Fetal Liver (FL): (*Left*) Gating on $CD45^{+}$ cells identifying Dendra-g^{+} MMCs in male and female fetuses of mC and mI dams. (*Middle*) Frequency of MMCs within $CD45^{+}$ cells. One-way ANOVA analysis is shown. (*Right*) Frequency of MMCs within $CD45^{+}$ cells in offspring spleens of previously infected dams segregated by sex. Two-way ANOVA analysis is shown. (mC M n=6, mC F n=3, mI M n=3, mI F n=4; data are from 3 independent experiments using 3-5 pregnant mice dams per group and 3-5 offspring per pregnancy.)

ANOVA adjusted P * <0.033, ** < 0.0021, *** < 0.0002, **** <0.0001

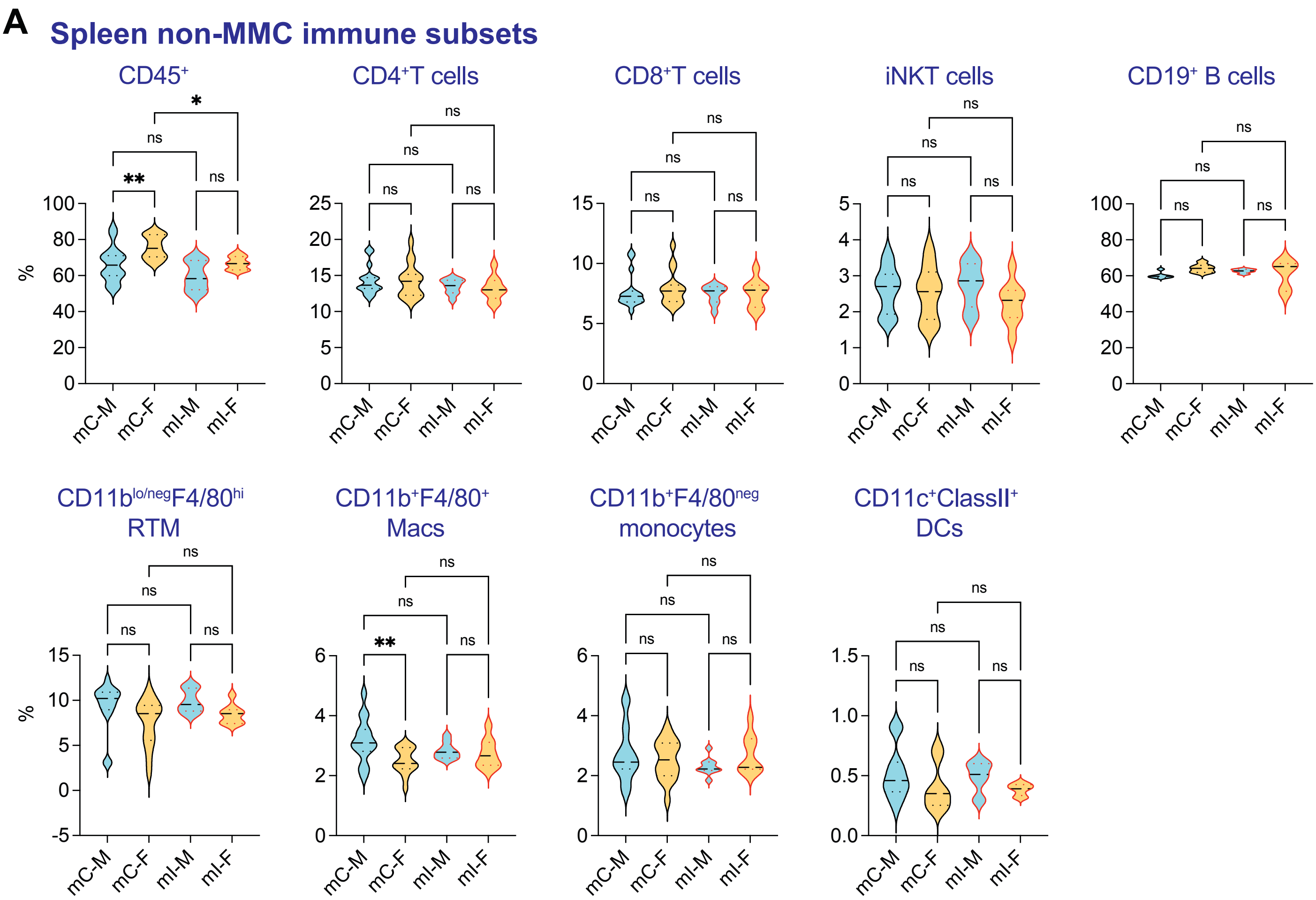

Supplemental Figure 4: Distribution of splenic (non-MMC) immune subsets in mC and mI offspring

Supplemental Figure 4: Distribution of splenic [non-MMC] immune subsets in spleen of PND28-35 offspring, related to Figure 2

Allogeneic matings of $Dendra^{HET}$ females with BALB/cJ males were set up to generate $Dendra^{WT}$ offspring. Timed-pregnant $Dendra^{HET}$ dams were orally administered *Y. pseudotuberculosis yopM* at embryonic day 7.5 (E7.5). Male and female $Dendra^{WT}$ offspring of infected (mI) and control (mC) mothers were analyzed at PND28-35.

(A) Frequency of $CD45^+$ cell, $CD4^+$T cells, $CD8^+$ T cells, iNKT cells, $CD19^+$ B cells, resident tissue macrophages (RTMs; $CD11b^{lo/neg}F4/80^{hi}$), macrophages (Macs; $CD11b^+F4/80^+$), $CD11b^+F4/80^{neg}$ monocytes and $CD11c^+ClassII^+$ dendritic cells. (mC male n=16, mC female n=16, mI male n=9, mI female n=9; data are from 5 independent experiments using 3-4 pregnant mice dams per group and 4-6 offspring per pregnancy)

One-way ANOVA adjusted P: ns not significant, * <0.033, ** < 0.0021, *** < 0.0002, **** <0.0001.

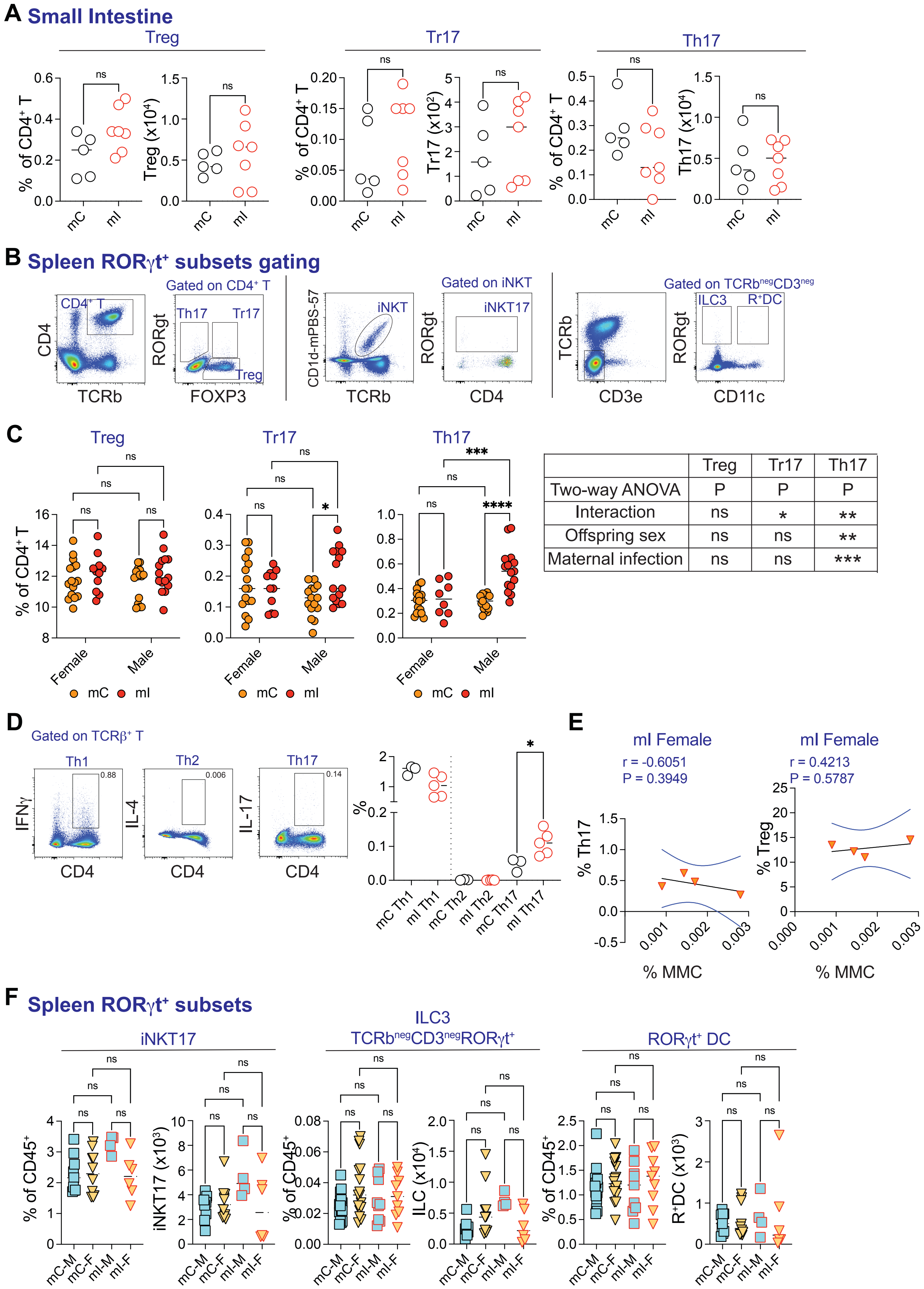

	Treg	Tr17	Th17
Two-way ANOVA	P	P	P
Interaction	ns	*	**
Offspring sex	ns	ns	**
Maternal infection	ns	ns	***

Supplemental Figure 5: Distribution of RORγt+ subsets in small intestine and spleen of mC and mI offspring.

Supplemental Figure 5: Distribution of RORγt expressing cell subsets in small intestine and spleen of PND28-35 offspring, related to Figure 2

Allogeneic matings of Dendra^{HET} females with BALB/cJ males were set up to generate Dendra^{WT} offspring. Timed-pregnant Dendra^{HET} dams were orally administered *Y. pseudotuberculosis yopM* at embryonic day 7.5 (E7.5). Male and female Dendra^{WT} offspring of infected (mI) and control (mC) mothers were analyzed at PND28-35.

(A) Small Intestine (SI): Frequency and numbers of (*From left to right*) Treg cells, RORγt^{+} Treg (Tr17) cells and Th17 cells. (mC n=5, mI n=7; data are from 2 independent experiments using 2 pregnant dam per group and 2-4 offspring per pregnancy; t-test P ‘ns’: not significant).

(**B) Spleen RORγt^{+} subsets gating strategy:** Representative flow cytometry dot plots show gating strategy to identify RORγt^{+} subsets including CD4^{+}RORγt^{+} Th17, CD4^{+}RORγt^{+}FOXP3^{+} Tr17, CD4^{+}RORγt^{neg}FOXP3^{+} Treg, CD1d-mPBS57-tetramer^{+}TCRb^{+}RORγt^{+} iNKT17, CD3e^{neg}TCRβ^{neg}RORγt^{+} ILC3 and CD11c^{+}RORγt^{+} DCs.

(**C) Spleen PND28-35:** Frequency of Tregs, Tr17 and Th17 cells in offspring spleens of previously infected dams segregated by sex. Two-way ANOVA analysis is shown (mC male n=15, mC female n=16, mI male n=15, mI female n=11; data are from 5 independent experiments using 3-4 pregnant mice dams per group and 4-6 offspring per pregnancy; two-way ANOVA adjusted P: ns not significant, * <0.033, ** < 0.0021, *** < 0.0002, **** <0.0001).

(**D**) (*Left*) Gating strategy for identifying CD4^{+}IFNg^{+} Th1 cells CD4^{+}IL-4^{+} Th2 cells and CD4^{+} IL-17^{+} Th17 cells. Splenocytes from C57BL/6 mice were stimulated with PMA + Ionomycin in the presence of Golgi Stop and Golgi Plug for 6 hours followed by intracellular cytokine staining. (*Right*) Frequency of Th1, Th2 and Th17 cells in splenocytes from mC and mI offspring (6-8

weeks old) after PMA+ Ionomycin stimulation. Data are representative of 2 experiments with 3-5 mice per group.

(E) Correlation analysis of the percentage of splenic MMCs versus the percentage of Th17 and Treg cells in female mI Dendra^{WT} offspring. The black line represents the linear regression, and the blue line indicates the nonparametric Spearman correlation with a 95% confidence interval.

(**F**) **Spleen PND28-35:** Frequency of iNKT17 cells, ILC3s and RORγt^{+}DCs in offspring of previously infected dams. (mC male n=15, mC female n=16, mI male n=9, mI female n=9; data are from 5 independent experiments using 3-4 pregnant mice dams per group and 4-6 offspring per pregnancy; one-way ANOVA adjusted P: ns not significant).

A Salmonella infection Day 5

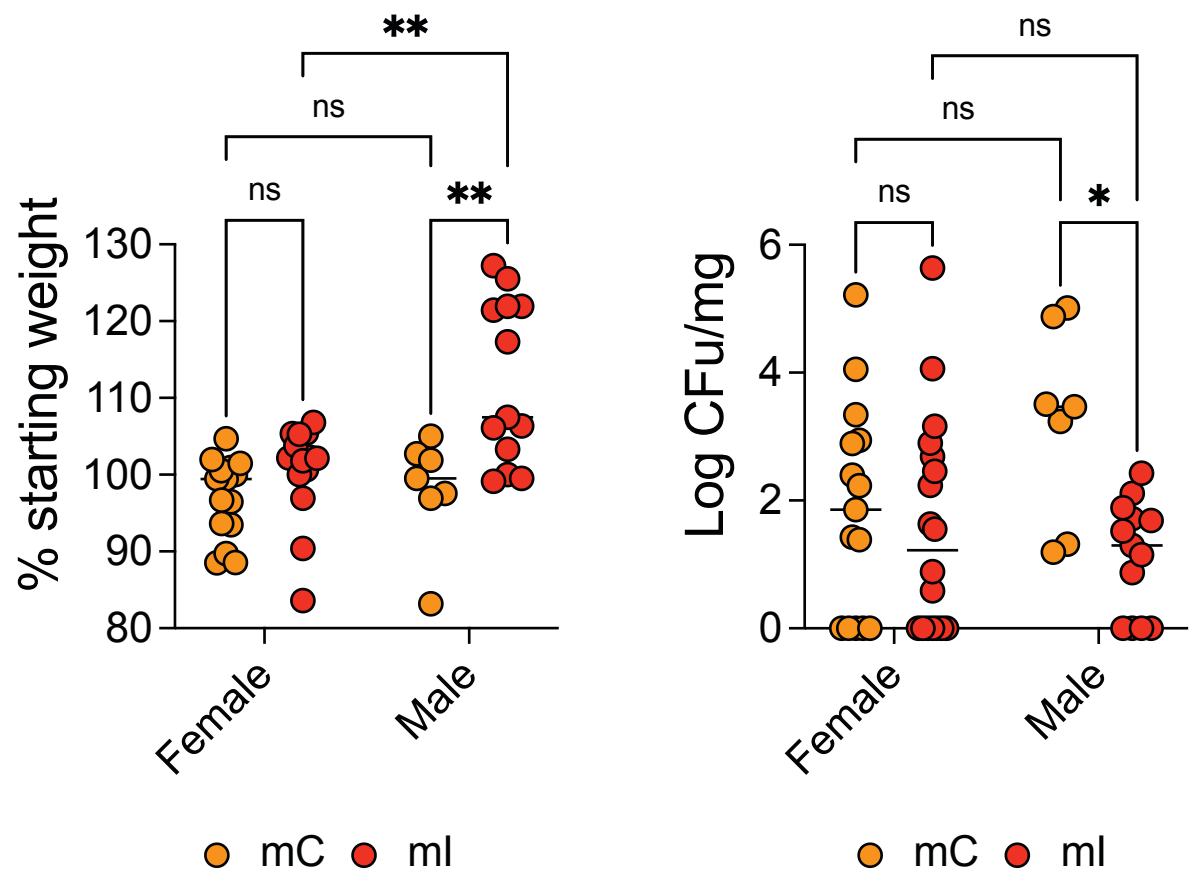

	Body weight	log CFU/mg
Two-way ANOVA	P	P
**Interaction**	*	*
Offspring sex	**	ns
Maternal infection	***	**

Supplemental Figure 6: Pregnancy-associated maternal infection increases resistance of male offspring to *Salmonella* infection

Supplemental Figure 6: Pregnancy associated infection increases resistance of male offspring to *Salmonella* infection, related to Figure 3

Dendra^{WT} offspring of uninfected (mC) or previously infected dams (mI) were orally administered *Salmonella* Typhimurium (SL1344 mutant) at PND28-35.

(A) *(Left)* Percent starting weight and (*Right*) CFU 5 days after *Salmonella* infection in offspring of previously infected dams segregated by sex. (mC-Salm M n=7, mC-Salm F n=15, mI-Salm M n=12, mI-Salm F n=18; data are from 4 independent experiments using 3-4 pregnant mice dams per group and 4-6 offspring per pregnancy; two-way ANOVA adjusted P: ns not significant, * <0.033, ** < 0.0021, *** < 0.0002).
