# Supplementary material for: Prenatal maternal infection promotes maternal microchimeric cells to alter infection risk in male offspring
Source: Front Immunol. 2026 Sep 3;17:1869048. doi: 10.3389/fimmu.2026.1869048 (PMC13582634; doi:10.3389/fimmu.2026.1869048)
Supplement: Supplementary file 2 [file Table1.docx]

**Table S1: Reagents List**

| **REAGENT or RESOURCE** | **SOURCE** | **IDENTIFIER** |
| --- | --- | --- |
| **Antibodies / Beads** |  |  |
| PLZF PE | BD Biosciences | Cat#: 564850 RRID: AB_2738984 |
| F4/80 PeCF594 | BD Biosciences | Cat#: 56513 RRID: AB_2734770 |
| FOXP3 PeCy7 | Thermo Fisher Scientific | Cat#: 25-5773-82 RRID: AB_891552 |
| Sca-1 APC | BioLegend | Cat#: 108112 RRID: AB_313349 |
| CD1d mPBS-57 APC |  |  |
| Ly6C APC | BioLegend | Cat#: 128016 RRID: 1732076 |
| LPAM-1 APC | BD Biosciences | Cat#: 562376 RRID: AB_11154232 |
| CD16/32 APCR700 | BD Biosciences | Cat#: 565502 RRID: AB_2739269 |
| MHC Class II A700 | Thermo Fisher Scientific | Cat#: 56-5321-82 RRID: AB_494009 |
| TCRβ A700 | Thermo Fisher Scientific | Cat#: 560705 RRID: AB_1727573 |
| CD11c APC780 | Thermo Fisher Scientific | Cat#: 47-0114-82 RRID: AB_1548652 |
| TCRβ APC780 | Thermo Fisher Scientific | Cat#: 47-5961-82 RRID: 1272173 |
| CD103 Pacific Blue | BioLegend | Cat#: 121418 RRID: 2128619 |
| CD4 BV421 | BD Biosciences | Cat#: 562891 RRID: 2739779 |
| Ter119 BV421 | BD Biosciences | Cat #: 563998 RRID: |
| CD90.2 BV480 | BD Biosciences | Cat#: 566082 RRID: AB_2744090 |
| CD11c BV570 | BioLegend | Cat#: 117331 RRID: AB_10900261 |
| Ly6G BV605 | BD Biosciences | Cat#: 563005 RRID: 2737946 |
| CX3CR1 BV650 | BioLegend | Cat#: 149033 RRID: AB_2565999 |
| c-kit BV711 | BioLegend | Cat#: 105835 RRID: AB_2565956 |
| RORgt BV786 | BD Biosciences | Cat#: 564723 RRID: AB_2738916 |
| CD45 BV786 | BD Biosciences | Cat#: 564225 RRID: AB_2716861 |
| CD3e BUV395 | BD Biosciences | Cat#: 563565 RRID: AB_2738278 |
| Live Dead Fixable Blue Cell Stain Kit | Thermo Fisher Scientific | Cat#: L23105 |
| CD45 BUV563 | BD Biosciences | Cat#: 612924 RRID: AB_2722550 |
| CD19 BUV563 | Thermo Fisher Scientific | Cat#: 365-0193-82 RRID: 2925369 |
| H-2Dd BUV615 | BD Biosciences | Cat#: 751198 RRID: 2875220 |
| CD4 BUV661  CD11b BUV737 | BD Biosciences  BD Biosciences | Cat#: 612374 RRID: AB_2870246  Cat#: 612801 RRID: AB_2738811 |
| CD8a BUV805 | BD Biosciences | Cat#: 612898 RRID: AB_2870186 |
| H-2DB BUV805 | BD Biosciences | Cat#: 748390 RRID: AB_2873074 |
| IFN-g BV421 | BD Biosciences | Cat#: 563376 |
| IL-4 APC | BD Biosciences | Cat#: 554436 |
| IL-17a PE | BD Biosciences | Cat#: 559502 |
| CD11b Microbeads | Milltenyi | Cat#: 130-097-142 |
| UltraComp eBeads Compensation Beads | Thermo Fisher Scientific | Cat#: 01-2222-42 |
| **Chemicals/Kits** |  |  |
| Brilliant Staining Buffer | BD Biosciences | Cat#: 566349 |
| Donor Bovine Serum | Thermo Fisher Scientific | Cat#: 10371-029 |
| FOXP3/Transcription Factor Staining Buffer Set | Thermo Fisher Scientific | Cat # 00-5523-00 |
| Fixation Buffer | BD Biosciences | Cat#: 554655 |
| BD Cytofix/Cytoperm | BD Biosciences | Cat#: 554714 |
| 1X Cell stimulation Cocktail (500X) | Thermo Fisher Scientific | Cat# 00-4970 |
| 1X Protein Inhibitor cocktail (500X) | Thermo Fisher Scientific | Cat# 00-4980 |
| Phosphate Buffered Saline | Thermo Fisher Scientific | Cat#: 20012-027 |
| Liberase TL Research Grade | Sigma-Aldrich | Cat#: 5401020001 |
| ACK Lysing Buffer | Sigma-Aldrich | Cat#: A10492-01 |
| DNase I | Sigma-Aldrich | Cat#: 10104159001 |
| DMEM | Thermo Fisher Scientific | Cat#: 21063029 |
| QIAmp Fast DNA Stool Mini Kit | Qiagen | Cat#: 51604 |
| Triton X-100 | Qiagen | Cat#: 51604 |
| Nuclease Free Water | Life Technologies | Cat#: AM9932 |
| HEPES (1M) | Thermo Fisher Scientific | Cat#: 15630-080 |
| 0.5 M EDTA, pH 8.0 | Thermo Fisher Scientific | Cat#: 15575-038 |
| SYBR Green (12X) | VWR | Cat#: 101414-16 |
| **Mice** |  |  |
| PhAM Excised Dendra C57BL/6J mice | The Jackson Laboratory | Strain#: 018397 RRID: ISMR_JAX:018397 |
| BALB/cJ mice | The Jackson Laboratory | Strain#: 000651 RRID: ISMR_JAX:000651 |
| **Bacteria / Primers** |  |  |
| *Yersinia pseudotuberculosis* 32777 (YopM) Strain | University of Pennsylvania (Dr. Igor Brodsky) |  |
| *Salmonella Typhimurium* SL1344 | Mass General for Children: Mucosal Immunology and Biology Research Center (MIBRC) (Dr. Bobby Cherayil) |  |
| *Yscf* forward primer | Integrated DNA Technologies | Ref#: 410636576 |
| *Yscf* reverse primer | Integrated DNA Technologies | Ref#: 410636575 |
| **Other** |  |  |
| YT Broth (2X) | Thermo Fisher Scientific | Cat#: 50-254-404 |
| MacConkey Agar | Thermo Fisher Scientific | Cat#: CM0007B |
| Agar | Thermo Fisher Scientific | Cat#: BP1423-500 |
| D(+) Glucose | Sigma Aldrich | Cat#: 50-99-7 |
| Percoll | Sigma Aldrich | Cat#: 65455-52-9 |
| MAC-1 Saporin | Integrated DNA Technologies | Cat#: IT-06 |
| Rat IgG Saporin | Integrated DNA Technologies | Cat#: IT-17 |
